# Goals of telephone nursing work - the managers’ perspectives: a qualitative study on Swedish healthcare direct

**DOI:** 10.1186/1472-6963-14-188

**Published:** 2014-04-24

**Authors:** Elenor Kaminsky, Marianne Carlsson, Inger K Holmström, Jan Larsson, Mio Fredriksson

**Affiliations:** 1 Department of Public Health and Caring Sciences, Health Services Research, Uppsala University, Box 564, SE-751 22 Uppsala, Sweden; 2 Department of Public Health and Caring Sciences, Caring Sciences, Uppsala University, Uppsala, Sweden; 3 Department of Health and Caring Sciences, University of Gävle, Gävle, Sweden; 4 Department of Public Health and Caring Sciences, School of Health, Care and Social Welfare, Mälardalen University, Västerås, Sweden

**Keywords:** Equitable healthcare, Health promotion, Managers, Paediatric health calls, Registered nurses, Sweden, Telephone nursing, Qualitative research

## Abstract

**Background:**

Swedish Healthcare Direct (SHD) receives 6 million calls yearly and aims at increased public sense of security and healthcare efficiency. Little is known about what SHD managers perceive as the primary goals of telephone nursing (TN) work and how the organisation matches goals of health promotion and equitable healthcare, so important in Swedish healthcare legislation. The aim of the study was to explore and describe what the SHD managers perceive as the goals of TN work and how the managers view health promotion and implementation of equitable healthcare with gender as example at SHD.

**Methods:**

The study was qualitative using an exploratory and descriptive design. All 23 managers employed at SHD were interviewed and data analysis used deductive directed content analysis.

**Results:**

The findings reveal four themes describing the goals of TN work as recommended by the SHD managers. These are: ‘create feelings of trust’, ‘achieve patient safety’, ‘assess, refer and give advice’, and ‘teach the caller’. Most of the managers stated that health promotion should not be included in the goals, whereas equitable healthcare was viewed as an important issue. Varying suggestions for implementing equitable healthcare were given.

**Conclusions:**

The interviewed managers mainly echoed the organisational goals of TN work. The managers’ expressed goal of teaching lacked the caller learning components highlighted by telenurses in previous research. The fact that health promotion was not seen as important indicates a need for SHD to clarify its goals as the organisation is part of the Swedish healthcare system, where health promotion should always permeate work. Time used for health promotion and dialogues in a gender equitable manner at SHD is well invested as it will save time elsewhere in the health care system, thereby facing one of the challenges of European health systems.

## Background

This study explores the possibility of combining national health system objectives of health promotion and equitable healthcare [1] with an efficient telephone healthcare service. It deals with how managers of the organisation Swedish Healthcare Direct (SHD) view the goals of telephone nursing (TN) work and whether health promotion and equitable healthcare are included in these goals. Health promotion refers to the process of planned actions that enable people to increase control over, and to improve, their health and well-being, physically, mentally and socially [2]. Equitable healthcare implies that people with the same needs should have the same services and access to healthcare, regardless of residence, gender, age, social group, and so on [1]. Since parent gender was recently found to play a role for the outcome in paediatric health calls [3], implying that sick children are not treated equally due to the gender of the parent making the call on their behalf, this aspect of equitable healthcare was used as an example in the study.

TN in Sweden is, as in many other countries, an expanding healthcare area, accessible on a 24/7 basis. The calls are free of charge, except for the cost of the call. Swedish healthcare is otherwise tax financed and patients pay a maximum yearly fee of approximately €120 (1100 SEK) for their healthcare. After 2013, when nationally completed, SHD is probably Sweden’s largest healthcare provider. Callers connect directly to telenurses at the nearest county or region through the national number, 1177. In total, there are 1100 employed telenurses at 33 workplaces. The telenurses are encouraged to keep calls 7–9 minute long [4] and use a decision support tool (DST) designed as a checklist based on caller symptoms [5]. Reported call outcome for self-care versus referral to other healthcare providers is approximately 50/50 [6-8]. Callers are however free to seek primary and emergency care irrespective of telenurse recommendations. The estimated number of calls is 0.6 per person and year for the 9.5 million Swedish inhabitants. This offers telenurses rich opportunities for health promotion as intended by the law. In paediatric health calls, which account for nearly half of the six million yearly SHD calls, investments of health promotion are likely to ensure long-lasting value. This, due to the young age of persons calls are made for (children age 0–17), facing a long life profiting from early health promoting activities.

TN is a special kind of healthcare as the nurses at SHD never see the callers [9]. The care is regulated in the same way as other forms of Swedish healthcare provisions, one consequence being that the nurses are not allowed to diagnose illnesses [10]. The most important regulations can be found in the Health and Medical Services Act [1]. For example, the law requires SHD to produce *“good health and healthcare on equal terms for the entire population”* and work to “*prevent ill health”*. The preventive aspect is also present in national guidelines for healthcare and in several other documents [11-15] with the central message that health promotion should systematically be integrated into all aspects of healthcare as a natural component in the chain of care.

The repeatedly mentioned objectives of SHD are to increase access to healthcare, increase citizens’ sense of security and increase the effectiveness of healthcare services [4,10]. The tasks for telenurses as described by SHD include to *’answer questions, assess care needs, give advice and refer callers to an appropriate level of care’*[10]. Yet, telenurses themselves describe their work to be more relational [16] and also mention supporting, strengthening and teaching callers, as well as facilitating their learning, which imply that they have a more comprehensive understanding of telephone nursing work. This last aspect of their work implies a potential for health promotion*,* provided that the caller receives self-care advice, one of the most common measures of health promotion [17], and not just a referral to another provider of care. In Sweden, mothers received twice as much self-care advice for their sick children compared to fathers, according to a recent study [3]. The discrepancy was not explained by any difference in the seriousness of the children’s condition, neither was it related to the child’s gender. Thus, contrary to the law’s intention as regards health promotion and equitable healthcare, health promotion is distributed unequally. If and how SHD intends to meet the requirements of the law remains unclear.

As a service, SHD is intended to lead to ’increased access to healthcare, increased public sense of security and an efficient healthcare service’ [4]. These organisational goals are likely to affect how managers view the goals of TN work [18]. The investment in SHD is part of the New Public Management (NPM) reform trend, associated with objectives of efficiency, cost control and performance evaluation. NPM objectives in healthcare have been linked to changes in the nurse manager role: from leading and supporting nurses who deliver care to policy implementation, quality measures, budgetary matters, etc. Because all calls are monitored, managers at SHD can supervise and control the telenurses’ work performance (length of calls, quality of the conversation etc.) in detail [19,20]. Thereby they can have a strong impact on the culture of for example health promotion [21]. The question whether telenurses are being encouraged to prioritise health promotion in situations of stress [22,23] and shortage of time [4] has thus far gone unanswered. Most of the managers are qualified registered nurses (RNs) and are hence concerned by, for example, the ICN ethical code for nurses [13] and the Swedish ‘Competence description for registered nurses’ [14]. Consequently, they may have difficulties in balancing these dual roles [24-26]. In practice, managers at SHD have to determine a way for the service to be efficient without losing caring aspects or long-term investments in callers (e.g., health promotion). Furthermore, according to Winblad [27], the extent to which health professionals take actions to fulfil political intentions such as systematic health promotion depends on whether they are 1) able to carry out the policy goals, 2) whether they understand the intentions behind the goals and the actual rules; and whether they are 3) willing to carry out the tasks to reach the policy goals. Whether they are able is e.g. conditional on the organisational structure and prerequisites for carrying out the policy; whether they understand e.g. on knowledge and interpretation of the policy; and whether they are willing on e.g. the correspondence to professional codes and ethics. The managers’ perceptions are discussed in relation to this tripartite model.

Against this background, this study aimed to explore and describe what SHD managers perceive as the primary goals of TN work and how they view health promotion and equitable healthcare implementation at SHD. Do the managers strive to match the legal goals of health promotion and equitable healthcare with SHD goals of efficiency and productivity?

## Methods

### Design

The study used an exploratory and descriptive design. A qualitative interview approach was chosen because of the character of the research question, with its aim to investigate the thoughts and experiences of a defined group of people [28,29]. The research conform to the RATS guidelines for qualitative research review [30].

### Procedure and study participants

All 23 managers employed at SHD were sent an e-mail with brief information about the study. Within two weeks the principal investigator (EK) called each manager to ask about participation in the study. All managers approved participation and were interviewed within the next three months.

The 23 managers were 21 women and 2 men in age 40–65 (M 54). They were at the time of the study responsible for SHD at Sweden’s 21 county councils and regions with 33 TN workplaces (32 in Sweden and 1 in Finland). One manager was responsible for three county councils. One region had five employed managers, whereof one was a coordinator. Twenty-one managers had a RN degree with a variety of specialties; their experience as nurses ranged between 9 and 36 years. One manager was a psychologist and one was a mental health worker. Three managers were privately employed and 20 were employed by the public healthcare system. The participants’ experience as SHD managers ranged from 0.3 to 12 years. Like most Swedish healthcare, the SHD sites were publicly financed. Sixteen managers had clinical experience in TN.

### Interviewing process

All interviews were performed by telephone by the first author over a 3-month period (March - May 2012). First, two pilot interviews, with a district nurse and primary healthcare teacher, and a manager in primary health emergency care, were performed via telephone. This was made to test interview questions and the telephone interview situation. The pilot interviews were transcribed and discussed among co-authors. After this, the interviews with each of the 23 SHD managers were performed, lasting from 35–70 minutes. The interview questions were semi-structured and focused on how the managers view the goals of TN work, health promotion and equitable healthcare implementation, see Table 1. The managers were asked about their views on telenurses’ understanding of work as described in a study of 2009 [16] and the unequally distributed parental result reported in a study of paediatric health calls of 2010 [3].

**Table 1:** Interview guide with interview questions asked in the study

1.	What do you, as manager, see as the goal for telephone nursing work, for example when parents call for their sick children?
2.	How do you view telenurses’ understanding of work, described in a study from 2009?
3.	How do you regard the Swedish public health goal of health promotion can be met through telenurses’ communication with parents in paediatric health calls?
4.	How do you relate your expressed goals for telephone nursing work and the Swedish public health goals to the telenurses’ requirements of efficiency and time limited calls in their work?
5.	What is your view regarding the mother caller percentage of 70 to 80 percent in paediatric health calls both in Sweden and internationally?
6.	In one of our earlier studies, mothers had received self-care to almost a double extent, compared to fathers, who instead, to the same extent received a referral to other health services. What is your view regarding this?
7.	If the above gender result was reproduced if measured today – how would you, as manager, deal with this?

### Ethical considerations

The present study followed the ethical regulations and guidelines according to the Swedish law [31], and conformed to the ethical principles defined in the World Medical Association Declaration of Helsinki [32]. All the managers gave their consent to participate after being informed about the study, that their participation was voluntary and that they were at liberty to withdraw from the study at any time. The participants were guaranteed confidentiality.

### Data analysis

The interviews were transcribed during and after each interview and subsequently analysed using deductive directed content analysis [33]. The starting point for this analysis was 1) what the managers described as the goals of TN work and if 2) health promotion and 3) equitable healthcare were included. To obtain a sense of the whole, interview transcriptions were read through several times by all authors. Text related to the study aim was highlighted, coded and distributed in the above 1, 2 and 3. The managers’ descriptions of the goals of TN work were then compared with how telenurses in an earlier study had described the core of their work [16]. The analysis was conducted by the first author, with all other authors acting as co-readers and discussed in research group seminars. Verbatim quotations in data that particularly summarized the managers’ opinions were chosen as illustrations in the finding section.

## Results

Four themes emerged from the SHD managers’ answers about what they conceive as the main goals of TN work: ‘create feelings of trust’, ‘achieve patient safety’, ‘assess, refer and give advice’ and ‘teach the caller’. Most of the managers stated that health promotion should not be included in the goals. By contrast, equitable healthcare was viewed as an important issue. The findings are presented in the following three domains: goals of TN work, health promotion, and equitable healthcare.

### The goals of TN work according to the managers

#### (a) To create feelings of trust

The managers expressed the need for telenurses to establish a relationship with callers, as a precondition for a smooth dialogue. Telenurses should strive to ensure parent satisfaction and security, working in a collaborative and supportive manner:

*‘The goal is … that parents are satisfied, that there is a feeling of reliance, that parents feel secure … that they are involved in the dialogue. Thus, to work together is the ultimate goal’*. Manager 9

To create feelings of trust and security reflects SHD’s general aim of ‘increased public sense of security’. The managers strongly believed that this would facilitate the parents’ delivery of information so the telenurses could more easily grasp the condition of a child and thus make correct assessments. Telenurses’ self-confidence was considered important in making concerned parents comfortable with the advice they had received:

*‘Many parents are worried,* [*so it is important] to make them comfortable with respect to the advice they receive’* Manager 16

#### (b) To achieve patient safety

Several managers described tools, such as the DST and the in-service trained ‘dialogue process’ to achieve patient safety as feature goals of TN work. The managers’ descriptions focused on structured work and quick assessment and the callers as persons were seldom mentioned. A few managers said that parents’ approval and understanding of what had been said during the calls could be checked to increase patient safety. This theme links to one of the SHD explicit work tasks, that telenurses should “refer callers to an appropriate level of care”. The available care levels telenurses can refer to are emergency care (‘highest level’), primary care (‘middle level’) and self-care in callers’ home (‘lowest level’). An appropriate level means the lowest (i.e. cheapest) possible effective treatment level. Telenurses’ self-care advice to callers is considered the least expensive available measure. Embedded in patient safety is the law requirement of treating everyone fairly and equitably.

#### (c) To assess, refer and give advice

To assess, refer and give advice to callers in need of support, explicit tasks of the SHD service, were frequently recurring topics in the managers’ descriptions of the goals of TN work*.* Self-care advice should be given in an easily graspable way, based on procedures from the DST:

*‘The basic goal of TN work … is to make an accurate medical assessment and from this give advice, whether it concerns self-care or whether you should seek treatment, and if so, how urgent it is’.* Manager 7

#### (d) To teach

To teach the caller was another theme the managers regarded as a goal of TN work. Yet, the managers’ descriptions of this mostly concerned teaching parents with sick children to seek care at appropriate level. Instructing parents about the care level difference of primary care versus children emergency department was reported to be an important subject of telenurses’ teaching. Some managers believed telenurses’ teaching might help parents in the future, at the next time of illness or for their next child. Whether the caller or parent had learned anything was, however, not discussed.

*’Education: to tell them what to do, to search primary care or ED’* Manager 13

Comparing the managers’ views on the goals of TN work with telenurses’ own ways of understanding their work as described in an earlier study [16], established two shared themes were: ‘assess, refer and give advice’ and ‘teach the caller’. Other aspects of work seen by the nurses – ‘support’, ‘coach’ and ‘facilitate the callers’ learning’ – were not expressed by the managers. On the other hand, two of the managers’ goals, ‘create feelings of trust’ and ‘achieve patient safety’ were not represented in the telenurses’ descriptions of the core of their work [16]. When managers were asked to comment on this, all, though, considered all the categories reported in that study as relevant.

### The managers’ views on health promotion

More than half of the SHD managers explicitly stated that health promotion is not included in the SHD commission. For example, one manager said:

*“We can’t exaggerate public health work. It’s wasting our resources”.* Manager 17

In contrast, a few managers instead emphasised that health promotion *could* indeed be achieved through telenurses work with callers:

*“I think it is actually what we should primarily work with here*”. Manager 15

The managers gave many examples of barriers to health promotion at SHD, such as the telenurses’ unawareness of the social determinants of health, lack of time, lack of support from the DST, and lack of continuity of care. The managers stressed that short questions about diseases were the most frequent ones and that the primary mission of SHD is to treat illness and disease. They stated that telenurses do not have time for health promotion because of long patient queues and limited time per patient. Still, some emphasised that there are no demands concerning time efficiency for telenurses at SHD:

*‘We don’t have any demands on the number of calls per hour. Actually, every call is a new working task, and sometimes one call takes longer time than another call. It is something of a lottery for the telenurses which call they happen to reply to’.* Manager 14

Despite the earlier description of self-care advice from the DST, the managers felt the DST lacked health promotion components and that focus was more on whether a child’s symptoms would require a doctor’s visit or not.

*‘Our computerised decision-support system does not support us in that* (health promotion) *discussion’*. Manager 19

According to the managers, because a different telenurse replies to every new call, the lack of continuity of care rules out the possibility to efficiently follow-up health promotion. Some managers reported that they encouraged those telenurses who showed individual interest in practicing health promotion. Examples given were when telenurses, in connection with vacation trip issues, recommended sun protection and explained the risk of dehydration. Other managers accepted telenurses’ individual attempts for health promotion, but would not openly encourage this behaviour:

*‘It is not that I go out and say, this is how you should work’.* Manager 12

Based on the managers’ responses, health promotion is performed sporadically, without orientation (e.g., in self-care advice). Yet, many managers questioned the expediency of health promotion in telephone calls. Aside from lack of continuity of care, some managers felt it was important not to make the caller feel offended or to provide callers with more information than they initially requested. Apparently, health promotion was not thought to increase parents’ feelings of trust. Furthermore, health promotion was considered to require a much earlier start than when a parent of a sick child calls SHD, i.e. it should be a part of primary care (e.g., a school health service or child welfare centre).

To summarise, based on the interview material, strategies for health promotion appear to be lacking in TN work. First, health promotion was reported not to be included in the SHD commission, and second, managers expressed few visions for health promotion but rather questioned the expediency of practicing it in TN work. The managers also stressed that to practice health promotion effectively, telenurses need further education.

In the paragraph to follow we will report about managers’ views on the influence of parent gender on paediatric calls [3], i.e. the fact that fathers call less often and that, when they do call, they receive less self-care advice and health promotion than mothers. Do managers believe they can influence the skewed parental distribution?

### The managers’ views on equitable healthcare

The managers believed equitable healthcare is an important issue. The fact that mothers make the majority of paediatric health calls was seen as a family issue and nothing SHD managers can do anything about. Most of the managers were nevertheless concerned about the finding that father callers were referred to other health services more often than mothers [3]. The managers’ reactions to these results varied, from some who were upset about the disparities to those who rejected the results as unreliable. Most managers believed father and mother callers continue to be treated differently. Drawing on their experiences from handling calls themselves and/or managing telenurses at the SHD sites, they gave diverse possible explanations for these results and had varying opinions regarding whether and how to achieve equitable healthcare at SHD*.* Their explanations to the unequally distributed results focused on the callers, i.e. the users of the service, and the telenurses and the organization, i.e. the professionals and the provider.

### Possible caller explanations

Fathers were considered more assertive and more authoritative, exaggerating symptoms more and avoiding telenurses’ questions and self-care advice. The managers reported that fathers tend to more directly ask for a referral to other health services (e.g., asking what emergency ward to visit). Such behaviour was believed to stem from fathers’ inexperience in describing symptoms and performing self-care, which make them worried and insecure:

*‘It may be the case that when fathers call, they are more assertive? Alternatively, they might be more worried because they lack knowledge? They don’t see the children when they are sick. Therefore, the fathers might think the illness is more serious than it really is, which makes them more assertive in their actions’.* Manager 4

The unclear position of the father was evident when a mother had asked him to call SHD despite the fact that the father had been at work all day and had no current information on the child’s condition. This could for instance happen when a mother had been denied referral from a telenurse. The mothers were reported to have received self-care advice more frequently because they describe symptoms more fully and tend to be more secure and patient during a call than fathers. Managers interpreted giving more self-care advice to mothers as a way of hindering them from being referred to a higher level of care, with telenurses acting as gatekeepers by placing mothers in a subordinate position (compared to fathers):

*’Fathers are being cuddled, while mothers are being obstructed. I have also noted that men talk differently with me. Women have a tendency to be more descriptive, whereas men have a tendency to describe things based on facts. ’* Manager 23

In spite of all this, the managers maintained that when a telenurse hears a mother saying that something is wrong with her child, such a report is taken seriously indeed.

### Possible telenurse/organisational explanations

Managers suggested, as an explanation of the gender imbalance, that telenurses, unused to calls from fathers, believe that fathers lack knowledge in self-care and thus need more extensive help at other health services. Therefore, contrary to the objectives of equitable healthcare, telenurses give different questions and answers to fathers compared to what they give to mothers. The increased frequency of father referrals was thought to be due to female telenurses taking fathers more seriously. Hence, according to the managers, telenurses give way to male arrogation to avoid unpleasantness and make fathers satisfied, instead of reassuring them and promoting their health knowledge.

The managers underscored that fathers’ concerns and need for reassurance are largely ignored. Female-to-female communication was deemed easier (e.g., discussing performance of self-care and its expected result). Following this line of thought, the managers suggested a patient safety aspect: if a child’s condition remains unclear because of incomplete communication, a referral could be a correct measure. Managers also believed inequity in society to be mirrored in the telephone health service sector.

Some managers avoided commenting on the unequally distributed results, emphasising that the DST, which makes no difference between fathers and mothers, is always used in children assessments. The reported gender imbalance had been discussed during a manager network meeting, with the conclusion that each call is unique, no matter who the caller.

### The managers’ suggestions on how to achieve equitable healthcare

Most managers suggested ways to achieve equitable healthcare in paediatric health calls. One proposal to enhance the number of calls from fathers was by exposing fathers more in advertisements for the SHD service. Which of the parents that calls SHD was defined as a family issue. Concerning the diverse outcomes in paediatric calls [3], managers said that telenurses need to reflect on this problem and should be made more aware of unequal treatment due to gender. The managers also reported a need to encourage telenurses to practice their professional knowledge and not accept non-professional decisions.

*‘We must look at what makes us give way to men … to permit someone without nursing education to decide.’* Manager 3

Another suggestion was to influence the telenurses’ preconceptions of mothers as being the most appropriate persons for caring for children. One proposed measure for change was to perform a random selection of daily calls and allow telenurses to reflect over them, observing the sender-receiver messages and asking themselves whether the outcome would have been different if a mother/father had instead made the call. A few managers suggested the need for telenurses to present fathers with more explicit questions, as well as to have the fathers explain themselves more fully. Another issue discussed was whether more male telenurses at the services would make a difference. Lastly, there was a desire to lift gender questions to a higher level (e.g., co-operation with a centre for gender in order to achieve a more long-term equitable telephone healthcare).

## Discussion

Of the SHD managers’ four perceived goals for TN work, ‘create feelings of trust’, ‘achieve patient safety’ , ‘assess, refer and give advice’ and ‘teach the caller’ , the last two are in good agreement with how telenurses describe their basic work. There seems hence to be a discrepancy between how the TN profession and the SHD managers view the core of, or goals of, TN work. The managers’ goals are congruent with the formal organisational goals of SHD, supplemented by the goal “teaching callers”. Telenurses go further than managers and propose that callers’ learning should also be a part of their work. Thus, telenurses include a mixture of visionary and operational goals [34], whereas mangers in this study focused primarily on operational goals.

In the discussion now to follow, about managers’ views on how to achieve equitable healthcare and health promotion, we will apply to the tripartite model presented in the introduction. It focuses on whether key stakeholders *want to* and *are able to* perform the relevant measures and whether they *understand* the purpose of the measures or how to take action regarding implementation of reforms or how to achieve certain goals [27].

The fact that most SHD managers stated health promotion should not be included in the goals of TN, implies that the intentions of the law [1] and other documents [11-15] about health promotion are not being met at SHD. One way to interpret managers’ lack of vision about health promotion could be that many of them do not *understand* the legal requirements and intentions of health promotion in Sweden. Only a few of the managers (and all of these had previous work experience in health promotion) stated that SHD should work more seriously in the area of health promotion. It is also possible that the managers are influenced by different interpretations of the law in the self-governing county councils and regions. The managers felt the DST was not supportive of health promotion, suggesting that the managers do not think that the SHD nurses *are able* to carry out health promotion. The managers did, however, note that the DST supported self-care advice, a common measure of health promotion [17]. The reason for this discrepancy might be that self-care is commonplace at SHD, whereas the concept of health promotion is used in a broader sense, leading to confusion as to what it actually means and how it can be practised [35]. Many of the managers’ descriptions suggest they do not *want to* perform health promotion because they feel this should be done within primary care. Consequently, these managers do not encourage the integration of health promotion in paediatric health calls.

Lack of time was looked upon as a barrier to the practice of health promotion, which is in accordance with previous findings [36]. This may be construed as another example of the difficulty for telenurses to carry out the process of health promotion within the existent time frames. For most managers, it is neither possible nor desirable to combine health promotion with the efficiency and productivity goals of SHD [4,37]. The calls should be short and deal with diseases only. At the same time, the telenurses, supposedly self-confident and caring, should create feelings of trust for their parent callers. Consequently, there is a conflict between SHD’s internal organisational goals of efficiency and SHD’s role in the Swedish healthcare system. The SHD managers’ descriptions reveal that SHD fails to take actions to fulfil legal requirements of Swedish healthcare, time being expressed as one of the barriers.

At the initiation of SHD in 2003, the official investigator, Swedin, suggested that a well-functioning telephone service can improve the efficiency of the healthcare system, primarily by controlling or relocating patient flow to the lowest effective treatment level and thereby reducing the number of unnecessary healthcare visits [4]. At the same time, it was noted that if resources are scarce, calls become forced and telenurses have little or no time for advice and caring, the efficiency potential will be reduced and callers will be exposed to medical risks. Thus, telenurses’ work with self-care advice and health promotion, although time-consuming, could be an investment with long lasting value for the Swedish healthcare system in general. Accordingly, telenurses should, for example, reassure concerned fathers in order to increase feelings of trust.

Although the managers *understood* equitable healthcare to be an important issue, the mother caller majority was considered a family issue and nothing managers thought they can influence. Managers stated they were not *able to* influence who takes on the responsibility to call in the family, and it was not clear whether they *wanted to* influence it. This corresponds to gender roles in the family and society [38,39]. Furthermore, it ignores telenurses’ reports that female callers are easier to communicate with and that parents seem to have a greater trust in a mother’s ability to communicate with telenurses [40]. Managers however expressed a desire to change telenurses’ preconception of fathers as the parent least appropriate for dealing with children. Such a change could inspire fathers’ continuation to call SHD, with a more equitable parental distribution as a result. The report of mothers receiving self-care advice to a higher extent and fathers being referred to other services to a higher extent [3], was unknown to most of the managers, who were however not surprised at these results. The managers seemed to rank self-care advice below referrals in the care level hierarchy, as noted by the way they talked of hindered mothers, and fathers being pampered by the system and taken more seriously, when being referred by telenurses. Perhaps, there is thus a need for upgrading and valuing self-care advice within SHD? It also indicates a need for increased knowledge and understanding of how gender norms are reproduced at SHD.

### Strengths and limitations

Two of the authors (EK and IH) have TN experience from contexts outside SHD, whereas the other authors (MC, JL and MF) do not, which allowed for two viewpoints (emic and etic). As regards trustworthiness [41], credibility was achieved by including all employed SHD managers in the study and by transparency of the data collection and data analysis process [42]. Dependability was achieved by presenting the findings with the respondents’ quotes and describing the research process [43]. Transferability deals with the extent to which the findings can be transferred or generalised to other contexts or settings [41,43]. The census sample of all 23 employed managers at SHD ensures transferability. This, as telephone health services in Western countries have much in common and belong to a rapidly expanding area of healthcare. Finally, confirmability was achieved because the findings were well grounded in data [43]. This was achieved by continuous discussions at research seminars. Data are self-reported and hence not describing how the managers actually carry out their obligations at SHD.

## Conclusion

This study underlines the necessity of specifying internal goals and how to meet the goals of the healthcare system as a whole when introducing a new healthcare provider such as SHD in a healthcare system. The managers at SHD generally agreed with the explicit goals of the organisation, but also mentioned teaching. Facilitating the callers’ learning, an important aspect of TN work, highlighted by telenurses, was, however, not mentioned at all by the managers. Further, health promotion, which should be included in all fields of healthcare according to Swedish regulations, is not part of the aims and principles of SHD at all. It could well be asked if the SHD leadership does not see their organisation as a part of the Swedish healthcare system. There is certainly a need for clarifying the specific goals of SHD and its telenurses. The argument that there is not enough time to practice health promotion should be looked upon critically: the consequence might be counterproductive to the efficiency goals of SHD in that callers will seek treatment elsewhere. Providing more time at SHD for health promotion and training telenurses to include it in the dialogues in a gender equitable manner, is instead likely to save time in other healthcare instances. This is perhaps one way to face the expressed challenges of European health systems [44]. Call audits as proposed by the interviewed managers could be one way to increase telenurse gender competence. Finally, the decision support tool, DST, needs to be further developed to support health promotion and equitable healthcare.

## Competing interests

The authors declare that they have no competing interests.

## Authors’ contributions

EK, MC and IKH were responsible for the conception and design of the study. EK performed the data collection. EK, MC, IKH and MF analysed the data and drafted the manuscript. All authors (EK, MC, IKH, JL and MF) participated in the interpretation of the findings, the revision of the manuscript, and the approval of the final manuscript version.

## Pre-publication history

The pre-publication history for this paper can be accessed here:

http://www.biomedcentral.com/1472-6963/14/188/prepub
